# Mixed Model Approaches Can Leverage Database Information to Improve the Estimation of Size-Adjusted Contaminant Concentrations in Fish Populations

**DOI:** 10.1021/acs.est.4c10303

**Published:** 2025-03-05

**Authors:** Emily Smenderovac, Brian W. Kielstra, Calvin Kluke, Thomas A. Johnston, Satyendra P. Bhavsar, Robert Mackereth, Stephanie Melles, Gretchen L. Lescord, Erik J. S. Emilson

**Affiliations:** † Great Lakes Forestry Centre, Natural Resources Canada, Sault Ste. Marie P6A 2E5, Canada; ‡ Ecometrix, Great Lakes Forestry Centre, Natural Resources Canada, Guelph, Ontario P6A 2E5, Canada; § Vale Living with Lakes Centre, Laurentian University, Sudbury P3E 2C6, Canada; ∥ Vale Living with Lakes Centre, Ontario Ministry of Natural Resources, Sudbury P3E 2C6, Canada; ⊥ Ontario Ministry of the Environment, Conservation and Parks, Toronto, Ontario M9P 3V6, Canada; # Centre for Northern Forest Ecosystem Research, Ontario Ministry of Natural Resources and Forestry, Thunder Bay P7B 5E1, Canada; ¶ Department of Chemistry and Biology, Urban Water Research Centre, Toronto Metropolitan University, Toronto M5B 2K3, Canada; ∇ Florida LAKEWATCH program, Forests Fisheries and Geomatic Sciences, University of Florida, Gainesville, Florida 32611, United States

**Keywords:** mercury, arsenic, fish, mixed effects models, contaminant modeling

## Abstract

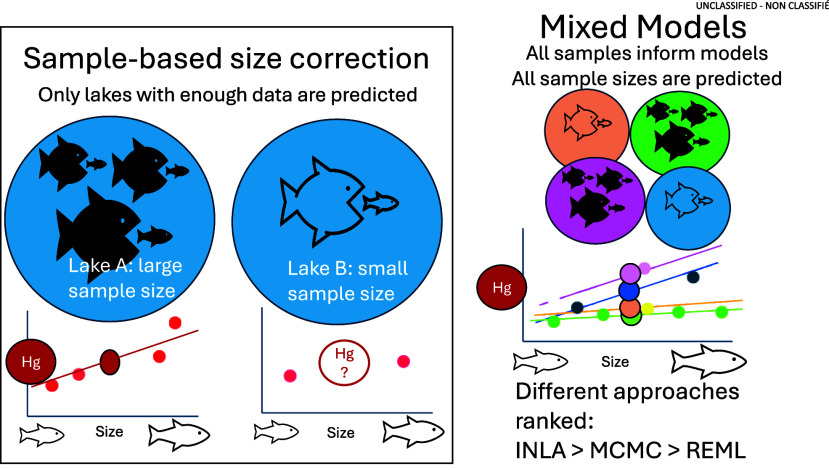

Concentrations of bioaccumulative contaminants in fish increase with their size and age; thus, research and monitoring of these contaminants in fish across space and time can be confounded by size covariation. To account for this, size-standardization of contaminant concentrations within fish samples is a common practice. Standardized concentrations are often estimated using within-sample regression models, also known as power series regression (referred to here as sampling event regressions, or SERs). This approach requires higher sample sizes than mixed effect models (MEMs), which are suited for this application but are not as commonly used. Herein we compare SERs to three MEM approaches; restricted maximum likelihood, Bayesian inference via Markov chain Monte Carlo (MCMC), and approximate Bayesian inference with nested Laplace approximation (INLA). We did this for two contaminants: mercury (Hg), a contaminant known to bioaccumulate, and arsenic (As), where the bioaccumulative potential is less understood. The MEM approaches generated size-standardized concentrations for small populations (e.g., <5 fish) and/or populations that lacked the range of sizes required for SER estimates, with comparable residual and root mean squared error to SER estimates. INLA was determined to be the best method in most cases because it was computationally less intensive than other approaches and showed consistent performance across a range of scenarios with sample-size limitations. Additionally, we provided example code for prediction using the R-INLA package to enable use and application in fisheries’ contaminant monitoring and research.

## Abstract 2

This work is relevant to ecotoxicology and contaminant research where contaminant concentration is often standardized to body size or age.

## Introduction

Older and larger fish tend to have higher concentrations of bioaccumulative contaminants that are assimilated faster than they can be excreted (e.g., mercury, organochlorides). Thus, contaminant monitoring programs often standardize concentrations of these contaminants to common ages, lengths, or weights across fish populations when making comparisons or dietary recommendations, such as in the Guide for Eating Ontario Fish.^1^ Standardization is also used to control for body size (or age) covariation when comparing contaminant concentrations among study sites (e.g., investigations into drivers of contaminant concentrations due to land use, forest change, and natural variation^2−5^) or among different time periods (e.g., temporal trend analysis).

A common approach for standardization of fish contaminant concentrations in both research and monitoring programs is to generate a linear equation between concentration and a body size metric (oftentimes, these relationships are power series regressions), and this equation is used to estimate the contaminant concentration at a chosen representative size (e.g., 500 or 1000 g) for each fish population.^1,4,6−8^ This approach (referred to here as sampling event regressions, or SERs) is applied to a broad range of monitored contaminants that can vary in bioaccumulative potential based on chemical characteristics, location, and fish species.^1^ The limitation of the SER approach is that it requires multiple fish (e.g., >5 fish/population^9^) across a suitable range of body sizes, which can be cost- or time-restrictive for monitoring or research programs. Smaller sample sizes can lead to poor inferences^10^ and/or exclusion of underrepresented waterbodies or species when using the SER approach.^11^

A potential alternative to SER, with fewer sample size limitations, are mixed effects modeling approaches. These approaches offer the advantage of “borrowing” information from other sampling events across an entire data set for to estimate overall “average” relationships while accounting for deviation in absolute values (i.e., random intercepts) or relationships (i.e., random slopes) among explanatory variables (e.g., waterbodies, times) to generate sample-level predictions and confidence intervals of contaminant concentrations for a given fish size.^12,13^ By borrowing strength from other observations, this approach can potentially increase accuracy and allow for estimates even when sample sizes are low or when there is an inadequate range of body sizes for the SER approach.

A likely barrier to the wide use of these mixed model methods, however, is the plethora of possible approaches, all of which have varied complexity, accuracy, and computational requirements. The differences in the error handling of these distinct approaches could affect their performance and sample size requirements. The most accessible implementation is the restricted maximum likelihood approach (REML), but it does not account for uncertainty in the random effects,^14^ which could reduce model fit and increase uncertainty in estimated concentrations. Relatively user-friendly, accessible statistical packages that facilitate the inclusion of confidence intervals around estimates are available.^15,16^ This includes bootstrapped likelihoods generated from REML or estimation of probability distributions either with a Bayesian Markov chain Monte Carlo (MCMC) approach or with the “approximate Bayesian” integrated Laplace approximation (INLA) approach. Briefly, these latter two approaches differ in how they estimate probability distributions: MCMC uses randomized resampling of both samples and model parameters,^16^ whereas INLA uses the (faster) Laplace approximations for probability distributions of each model parameter.^15,17^

Herein we compare four statistical approaches to determine the most accurate way to perform contaminant concentration standardization by fish body size, particularly under data limited scenarios. More specifically, we compared the commonly applied SER to four mixed effects model approaches that differ in how they estimate posteriors: (1) an REML approach, (2) an REML approach with bootstrapping (boot_REML), (3) a common Bayesian inference approach (MCMC), and (4) an approximate Bayesian inference (INLA) approach. We evaluated these approaches using two inorganic contaminants of concern in fish tissue that differ in bioaccumulative potential: mercury (Hg) and arsenic (As). We hypothesized that mixed model approaches would provide comparable or more accurate predictions of contaminant concentrations relative to SERs and that mixed models would still allow for estimation in data-scarce populations (i.e., when sample sizes are low and/or when data are limited across size ranges). Among mixed model approaches, we expected Bayesian approaches (i.e., MCMC and INLA) to have higher predictive accuracy than REML approaches because of the inclusion of uncertainty in random effects. We expected the REML_boot model to have improved prediction compared to the REML models for the same reason, though with less improvement than Bayesian approaches as bootstrapping is a more “brute force” approach to error estimation.

## Materials and Methods

### Data

#### Fish Contaminant Data

We obtained a data set containing total Hg and As concentrations ([Hg] and [As] respectively) in fish muscle and fish attribute data (lengths, weights, species) from the Ontario Ministry of Environment, Conservation, and Parks (MECP)^18^ and, from data collected in Northern Ontario for Arsenic studies.^3^ Although individual fish sampling protocols varied, muscle tissue sent to MECP was analyzed using standard methods as part of the Fish Contaminants Monitoring Program (FCMP).^18^ More specifically, total [Hg] was measured using cold vapor-flameless atomic absorption spectroscopy (CV-FAAS) following protocol OMECP-HGBIO-WS057 and total [As] was measured using inductively coupled plasma mass spectrometry (ICP–MS) following method OMECP-BIOTA-E3461. Arsenic data were collected as part of a study by Lescord et al.^3^ The As study was conducted at the ISO 17025-accredited Biotron trace-metal laboratory at the University of Western Ontario using the EPA method 3052 and 200.8 with microwave digestion and ICP–MS.

We included Hg because it is a highly bioaccumulative contaminant of global concern that is routinely monitored in fish and used to inform consumption guidelines.^19^ Size adjustments of [Hg] are also common using regression (i.e., SERs) to account for [Hg] variation across environmental gradients in research.^3,7,20−22^ We include As a representative contaminant that has also been size-adjusted in research,^3,23^ but is less bioaccumulative in fish, showing mixed or weaker relationships with metrics of body size.^3,23,24^ We further limited data to only inland lakes (i.e., excluding the Laurentian Great Lakes) and to three fish species: *Salvelinus namaycush* (Common Lake Trout, hereafter Lake Trout), *Esox lucius* (Northern Pike), and *Sander vitreus* (Walleye). These species were selected because while they are all predators, they represent distinct niches within lakes, and they are commonly consumed by anglers, so accurate estimation of contaminant concentrations is important for assessing human health risk. Our final data set included [Hg] in 37923 fish and [As] in 1001 fish. The data sets had a normal distribution of values, with fish between 400 and 1100 g_wet weight_ being well represented in the data (Figure S1). Generally, most fish in the data set had contaminant concentrations <1.3 μg/g_wet weight_ [Hg] and <0.4 μg/g_wet weight_ [As] (Figure S1).

#### Test and Training Data Sets

To facilitate the comparison of standardization approaches, we randomly sampled the fish contaminant data sets to create test and training subsets. We focused on the ability to predict concentrations for two common weight standardization targets of 500 and 1000 g_wet weight_, as these were round numbers well represented in the data set and commonly used in research studies.^3,6,7^ As there were few fish that are exactly 500 and 1000 g in the data set, but many fish surrounding these sizes, we decided to evaluate the predictive power for these sizes by evaluating the predictive accuracy for fish of similar sizes. As such, we randomly sampled and assigned 50% of contaminant data from fish weighing 500 ± 100 g and 1000 ± 100 g to the test data set, with the remaining 50% of data in those ranges and all data outside of those weight ranges assigned as a training data set (Table S1, Figures S1 and S2).

### Standardization Approaches

#### Sampling Event Regressions (SER)

For each sample of fish representing a unique contaminant–species–waterbody–year combination (i.e., a sampling event), we developed log-contaminant concentration (in μg/g_wet weight_) by log-wet weight (in g) regression models. SERs were limited to cases where the range of fish sizes allowed interpolative prediction (i.e., training sets needed to include values outside of the 400–500 or 900–1100 g test set ranges). Our data filtering may have been more lenient but also less subjective when compared to methods used in research, where researchers may curate data and remove outliers from each lake regression on a case-by case basis. Each model used the linear equation outlined in eq 1.
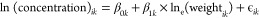
1where *i* is an individual fish for each species–waterbody–year combination event *k*, β_0*k*_ is the intercept, β_1*k*_ is the slope, and ϵ_*ik*_ is the residual error; these models were run on training data to create models for all species–contaminant–waterbody–year combinations in the study.

#### Mixed Effects Models (REML, boot_REML, MCMC, and INLA)

For each species and contaminant combination, we developed log–log [log-contaminant (μg/g_wet weight_) by log-g_wet weight_] linear mixed effects models including all waterbodies and years. We allowed for random variation in the slope and intercept per waterbody and random variation in the intercept by sampling event (i.e., each unique year and waterbody combination). The general model structure was as in 2.
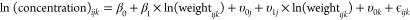
2where *i* is an individual fish in waterbody *j* during sampling event *k*, β_0_ is the fixed intercept, β_1_ is the fixed slope, υ_0*j*_ is the random intercept for waterbody *j*, υ_1*j*_ is the random slope for waterbody *j*, υ_0*k*_ is the random intercept for sampling event *k*, and ϵ_*ijk*_ is the residual error. This identical model structure was fit in all three mixed effects model approaches described below.

The REML models were fit using the *lme4* v1.1.34 package *lmer* function with default settings^25^ and the boot_REML analysis was performed using *lme4*’s *bootMer* function for parametric bootstrapping using 2000 simulations and with the use.u setting set to TRUE to simulate spherical random effects using 4 CPUs. The MCMC models were fit in STAN v2.32.2 through *rstanarm* v2.32.1. All models were run with 4 chains (and 4 CPUs)^16^ and the priors were the coefficients of an equivalent generalized linear mixed effects model. For [Hg], we used the default settings of 2000 iterations per chain and the adapt_delta setting of 0.8. For [As], however, the lower sample sizes required 9000 iterations and an adapt_delta setting of 0.99 for model convergence (at the cost of computation time). The INLA models were run in the *R-INLA* package with the “iid2d” model structure to account for covariance of the random effects due to waterbody.^26^ For REML models, the best linear unbiased prediction can be interpreted as an approximation of the mean, and for the bootstrapped REML, INLA, and MCMC predictions, we used the median derived from predicted test value distributions. Predictions were back transformed to concentrations using the exp function in R, to compare to measured values, and also to calculate root mean squared error (i.e., RMSE) for all models.

#### Population Medians (No Standardization)

We calculated the population median of [As] values for each of the three fish species individually from the training data of each waterbody–year combination (i.e., a sampling event) as it is possible that there is no bioaccumulation of this contaminant in some species. We included these results to provide context about the bioaccumulation of As and how that may have affected the model performance. We evaluated whether the median concentration was an accurate predictor of test values to demonstrate whether there was no arsenic bioaccumulation. We did not include this comparison for [Hg], as the bioaccumulation of Hg in these species is well documented while there has been some evidence that the long-term bioaccumulation potential of arsenic is low in the species in our study.^3,23,27^

#### Comparing Model Fit and Predictive Accuracy

To assess the predictive accuracy of each approach, we compared the back-transformed predicted concentrations versus observed concentrations for individual fish from the test data set using the linear relationship of the values with the *lm* function in R and, Pearson correlation with the *cor.test* function in R. We also assessed the fit of each approach by calculating the RMSE of the training data set for each sampling event (i.e., distinct year–waterbody combination). Slopes, intercepts, and model fit based on test data reflect the generalized (i.e., out-of-sample) predictive capability of a model (i.e., accuracy), while correlation values reflect the variation in accuracy for each concentration that is being predicted (i.e., precision). We used a threshold of 5 fish per sample event to separate low-sample number events from high sample number events in predictive comparisons as this threshold has been used in other studies to define suitable dataset sizes for SER.^23^

#### Simulation of Different Sampling Scenarios

We ran an additional analysis to assess how mixed model performance changes with different fish and lake number sampling scenarios using each year–waterbody as distinct sample events. We trained REML and INLA models on randomly sampled training sets with a range of lake numbers and fish numbers to evaluate the model predictive performance. We used the less computationally intensive INLA approach to represent both Bayesian approaches, as we determined over the course of this study that INLA and MCMC were highly similar in terms of predictive capability. This was run for a range of numbers of lakes (2, 3, 5, 7, 9, 10, 15, 20, 30, 40, 50, 75, 100, 150, 200, 250, 300, 500, 750, 1000) and a select number of fish sample sizes for each lake (3–20, 25, 30, 35, 40, 45, 50), with 10 replicate samples for each combination. We assessed the performance of these models by evaluating the trends of their slopes, intercepts, and Pearson correlation scores as the number of fish or the number of sample events increased.

#### Relative Computational Requirements

Computational times were assessed in the context of analyses performed in R version 4.3.1 (2023-06-16 ucrt)^28^ on a Dell Latitude 5510 PC running Windows 10 Enterprise with a 1.70 GHz, 2208 MHz 4 Core(s), 8 logical processor, 16 GB of physical memory and 34.6 GB of virtual memory. All data transformations, summarizations and graphing were performed with the *tidyverse* v2.0.0, *ggplot2* v3.5.0 and *ggpubr* v0.6.0 packages.^29−31^

## Results and Discussion

Of the approaches tested, REML and INLA were the most practical for producing accurate size-standardized estimates of the contaminant concentration for distinct waterbodies. Both techniques allowed for predictions in waterbodies where SER could not be performed. The predictions were highly comparable to measured values (linear associations of predicted and measured values approaching a 1:1 relationship and *r* > 0.75) and had favorable computational speeds when compared to other approaches. Setting aside inferential differences, REML may be more appropriate for many situations, as the implementation was less conceptually complex and more easily programmed at the time of writing this paper. However, INLA performed effectively on all data sets and had more predictable performance when run on a variety of simulated sampling scenarios.

**Figure 1:**
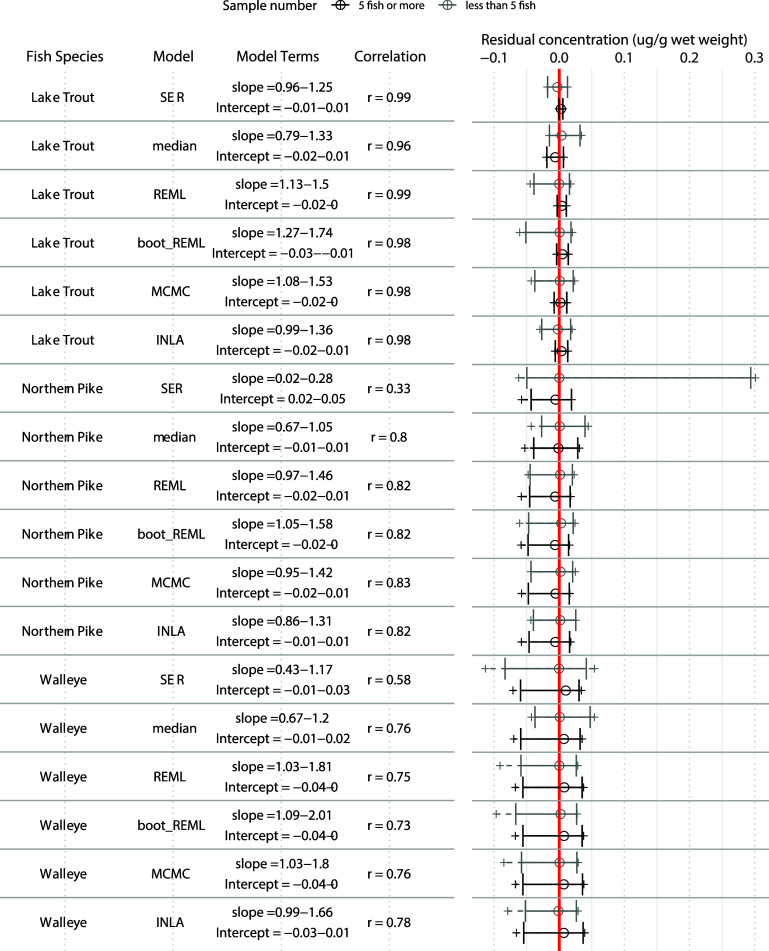
Comparison of predicted vs observed relationships for muscle [As] using predicted test-set values generated from six models. Predictions were made for 500 ± 100 g and 1000 ± 100 g fish of three species. The 95th confidence interval of slopes, intercepts, and correlation statistics (Pearson’s *r*) from linear association of predicted and measured values are indicated for each model. For correlations, values closer to 1 mean that values represent the trends of increases and decreases well. For slopes, values closer to 1 represent stronger representations of actual values when intercepts are close to zero. Residuals are plotted for events with less than five fish (gray), or 5 or more fish (black). A central hollow circle designates the median, with a solid error bar designating the 2.5, and 97.5th percentiles of the distribution. A dashed line ending in a + is used to show the range of remaining residuals. A vertical red line is used to highlight where the distributions overlap with zero.

**Figure 2:**
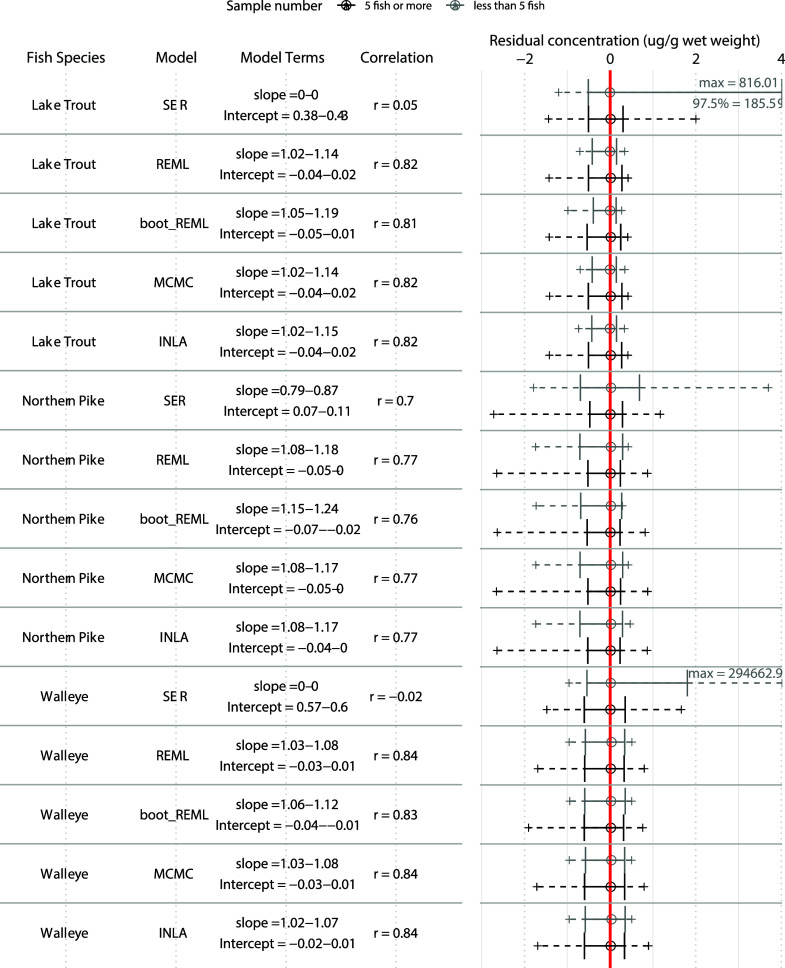
Comparison of predicted vs observed relationships for muscle [Hg] using predicted test-set values generated from six models. Predictions were made for 500 ± 100 g and 1000 ± 100 g fish of three species. The 95th confidence interval of slopes, intercepts, and correlation statistics (Pearson’s *r*) from linear association of predicted and measured values are indicated for each model. For correlations, values closer to 1 mean that values represent the trends of increases and decreases well. For slopes, values closer to 1 represent stronger representations of actual values when intercepts are close to zero. Residuals (predicted – observed) are plotted for events with less than five fish (gray), or 5 or more fish (black). A central hollow circle designates the median, with a solid error bar designating the 2.5 and 97.5th percentiles of the distribution. A dashed line ending in a + is used to show the range of remaining residuals. A vertical red line is used to highlight where the distributions overlap with zero. Particularly large outliers or percentiles are indicated in the text on the right side of the plot.

**Figure 3:**
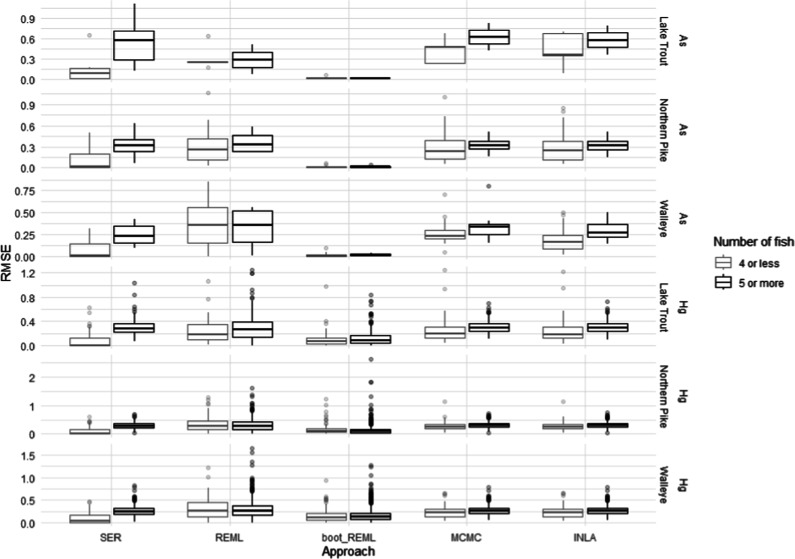
Root mean squared error (RMSE) from sampling events trained with different numbers of fish. RMSE of training set sampling events are compared to the number of fish used from the sampling event that were included in the training data set. For events with less than 5 fish, and events with 5 or more fish, a boxplot displays the 25th and 75th percentiles and the median RMSE, with the whiskers extending out to 1.5× the interquartile range. Higher RMSE means there was more variation in the data overall, and a wider box means there was more variability in the fit of predicted training values between distinct waterbody–year sample events. The figure shows data for [As] and [Hg] Sample event regressions (SER), restricted maximum likelihood (REML), REML with bootstrapping (boot_REML), Markov chain Monte Carlo (MCMC) and integrated Laplace approximation (INLA) models.

We found that mixed effects approaches performed better than SER as represented by improved slopes and intercepts, tighter residual distributions, and improved correlations. The mixed models also enabled predictions of values for low sample sizes with only slight accuracy reductions in the [As] models or marked accuracy improvements in the [Hg] models, compared to SER ([As] Figure 1, [Hg] Figures 2 and S3). Prediction accuracy of [Hg] with the REML, MCMC, and INLA (slopes deviating 0.02–1 from 1) approaches were better than SER (slopes deviating 0.13–1 from 1), but [As] prediction in REML, MCMC, and INLA (slopes deviating 0.01–1.01 from 1) were slightly poorer, compared to SER (slopes deviating 0.04–0.99 from 1). The reduced prediction accuracy in [As] was small, with slopes within 0.05 of SER models. The REML, MCMC and INLA values were generally good at representing the different concentrations in the data set, as they were more strongly correlated to actual concentrations (*r* ranging from 0.73–0.99 in [As] and 0.76–0.84 in [Hg]) than predictions from SER models (*r* ranging from 0.33–0.99 in [As] and 0.02–0.7 in [Hg]) (Figures 1, 2, and S3). The stronger correlations indicate that there is less variation in predictive accuracy for each concentration. The reduction in [As] mixed model performance may have been due to the lower overall data set sizes for the [As] models and were possibly also influenced by the assumptions of relationships between weight and [As] made in the structure of the mixed models. As [As] was more accurately predicted using medians for Northern Pike and Walleye than any of the linear modeling approaches, the assumed log–log relationship in the model structure was likely incorrect for those two species. Lake Trout might be an exception from this, as it could be prone to slightly more bioaccumulation of As due to its slow growth rate. The increased accuracy of [Hg] models can be attributed to the elimination of influential residuals that resulted from the magnification of error after the back-transformation of the log-transformed SER predictions. The residuals between the back-transformed predicted values and measured concentrations for the different mixed modeling approaches were generally similar to or smaller than those of the SER approach for both [As] (Figure 1) and [Hg] (Figure 2) models. Test/train sampling caused some prediction bias in both [As] and [Hg] data sets, some models had predicted–measured relationships that deviated from a 1:1 due to the presence of some influential prediction residuals from low-sample number prediction events. There were larger residuals influencing these relationships in [Hg], as there was a higher range of values than [As] across sizes (Figure S1), and thus more opportunity for larger deviations of rare test values from training values and, as a result slopes that did not overlap with 1. Interestingly, attempts to improve the REML approach with bootstrapping did not increase the accuracy of predictions, even resulting in larger deviations of slopes from a one-to-one relationship in some cases and decreased correlation strengths {specifically for the [Hg] Northern Pike and Lake Trout models (Figure 2), and the [As] Walleye model (Figure 1) ([As])}. This is likely because the bootstrapping process overfitted the models, as demonstrated by decreased RMSE (Figure 3), which reduced the ability of the models to produce generalized predictions.

All of the mixed model approaches effectively increased the number of sample events that could be used for prediction. Mixed model approaches were just as effective in lakes with <5 fish as they were in lakes with larger numbers of fish. This was demonstrated by the similar residual distributions of small sample events (<5 fish) and large sample events (>5 fish) by the mixed model approaches (Figures 1 and 2). SER was limited compared to mixed model approaches, as it does not borrow information from other samples, resulting in inaccurate predictions for low-sample numbers. The ability to create predictions for sample events that do not fulfill the requirements of SER is the main benefit of taking a mixed-model approach, though the mixed model approach also improved prediction for the highly bioaccumulative contaminant (i.e., [Hg]). In this study, the accuracy of mixed model approaches were always comparable to or better than SER or basic population medians so there does not appear to be a trade-off of accuracy for the prediction of additional, low-sample number sample events. The mixed model approaches allowed for the inclusion of sample events with less than 5 fish, resulting in a higher number of total sample events and individual fish concentration predictions, compared to SER (Table S1).

Differences in the RMSE for training data of the different sampling events illustrated the amount of variance that each model was trained to explain. INLA and MCMC models were more tightly fitted to the training data for the distinct sampling events, shown by lower RMSE, with fits comparable to those of SER (Figure 3). Conversely, REML approaches generally had distributions with a wider range of RMSE, including higher RMSE points for various sample-sizes. These highly influential values indicated that the patterns in some sampling events were more poorly represented than others in the global model. Boot_REML models often had the highest fits to the training data with lower RMSE than the other mixed model approaches or SER, but this did not correspond to the best predictive accuracy. This is likely due to bootstrapping procedures overfitting results to the training data, which reduced the extrapolative predictive capability of the models.

INLA and MCMC approaches had the best fits for low fish sample numbers, with fewer influential points than other mixed model approaches or SER. There was a trend among the models that low sample number events had lower RMSEs, likely because fewer values were fit and therefore overall variation was reduced. All approaches had RMSE that generally increased and stabilized at about 6 fish per sampling event but models were fairly comparable at event sizes below 6, with the exception of some events with influential points (Figure S4). These results suggest that, while higher sample numbers and size range distributions are always preferable, databases containing individual samples with as little as 6 fish may be able to be used to develop concentration predictions using mixed-model approaches. This information may be helpful for developing guidelines for field-based logistical decisions where remote access, sampling difficulty, or expense may conflict with sample number objectives. MCMC and INLA generally appeared to have fewer large influential RMSE values and a more even distribution of RMSE. These approaches were not restricted to minimum sample sizes and they had improved precision and model fit, given they could account for variation in lake-level slopes and intercepts.^8,32−34^

INLA and MCMC models had a good model fit, without a detectable loss in accuracy, making them suitable for understanding the mechanisms of prediction. Understanding the model fit of predictive approaches is not always necessary to ensure predictive accuracy, as higher fits can be higher due to exclusion of influential values or lack of balance in a data set. However, many researchers are interested in the relationships involved in these predictions. Size–concentration relationships can provide insight into bioaccumulation differences between waterbodies. The improved fit provided by the INLA and MCMC approaches showed that they were better at explaining the patterns in the training data than REML, and the improved fits had little or no cost to predictive accuracy (i.e., the models are better at predicting the general population trends and individual values). Thus, if a researcher is interested in understanding the mechanisms of prediction, the Bayesian approaches would be suitable options because they provide a more thorough evaluation of the selected data set. The generation of posterior distributions reduces the influence of individual influential values on researcher conclusions. INLA would be recommended for smaller data sets, as there was marked improvements of the INLA approach compared to MCMC on the smaller [As] data sets.

While our results demonstrated that Bayesian models could be used to create predictions of contaminant concentrations in fish for lakes with low sample sizes, these approaches are more complex and, thus, can have higher computational requirements. Computation times ranged from less than one s to 47,248 s (13.1 h) across modeling approaches and species. Overall, INLA models were much less computationally intensive compared to the MCMC and boot_REML implementations (Table 1). While REML was, generally, the quickest of all mixed modeling approaches, INLA was a close second, with computational times that were orders of magnitude lower than those of boot_REML or MCMC (Table 1). Researchers may opt to use MCMC over INLA, despite the additional computational requirements if they are interested in investigating the posterior distributions of parameters or predictions. INLA, being an approximate Bayesian approach, may be less adept at simulating the probability distributions compared to the repeated sampling approaches that are employed in MCMC. However, for predictive purposes, the overall accuracy of the INLA approach, when compared to the boot_REML approach, and paired with its improved speed compared to the MCMC approach suggests that INLA was the most viable option for standardizing fish contaminant values.

**Table 1:** Computational Time (in Seconds) for Each of the Five Modeling Approaches for Each Species and Contaminant Combination

contaminant	species	SER	REML	boot_REML	MCMC	INLA
As	Lake Trout	0.07	0.20	50.39	322.53	7.13
As	Northern Pike	0.38	0.39	57.10	3075.72	2.46
As	Walleye	0.38	0.33	43.90	2430.68	2.75
Hg	Lake Trout	3.37	0.94	326.28	10,722.27	5.40
Hg	Northern Pike	7.69	1.59	872.42	27,989.16	12.64
Hg	Walleye	8.07	1.78	750.76	47,248.49	14.51

Our evaluations of different sampling scenarios of varied numbers of lakes and fish numbers per sample event (see Materials and Methods: Simulation of Different Sampling Scenarios) confirmed that INLA and REML were effective at creating predictions for lakes with low sample numbers but both required a large number of lakes. Northern Pike, Lake Trout and Walleye [Hg] prediction was possible in simulations where 4 fish were used, but these predictions required more than 100 lakes (Figures S8, S9, S10, S14, and S15). For example, prediction of Lake Trout [Hg] required models with over 200 fish to predict using four fish per lake to produce predictions that were accurate (i.e., had higher correlation and approached 1:1 linear relationship with measured values with a zero intercept) (Figure S9). Arsenic results were more varied, with much poorer linear associations between predicted and measured values and poorer correlation strengths than [Hg]. However, none of the [As] data sets had a very large number of lakes, which limited this line of investigation. These results show that prediction of contaminant concentrations with few fish per sample event is possible when larger, existing data sets are leveraged. We suggest that creating predictive models that leverage the power of existing large data sets with hundreds of lakes, such as the MECP database used in this study, would be the most effective way to employ mixed-model standardization approaches.

The simulated samplings also revealed that REML models perform better with more balanced sampling designs than the INLA models. Generally, in the random samples, which had an equal number of fish selected for each lake sample event included in the sample, REML performed better than INLA models in correlations as well as prediction accuracy, as represented by the linear relationship between predicted and measured values approaching a 1:1 relationship (Figures S5–S16). This contrasted with the results from the assessment of the full test data set, where REML had greater or similar slope and intercept deviations from 1:1 relationships and similar correlation scores compared to INLA models (Figures 1 and 2). As the REML models had slightly lower performance in models created from the original test data set (Figures 1 and 2), this suggests that the REML models are more sensitive to sampling imbalance than INLA models. These results suggest that INLA would be preferred over REML in scenarios, for example, where researchers fail to collect sufficient numbers or size ranges of fish for each sample event, thus resulting in imbalanced data sets. In scenarios where there are balanced sample collections and a high number of sample events, the easier to implement REML models are likely to perform comparably to INLA. Both INLA and REML were biased toward slight underestimation concentrations, as evidenced by accuracy models with slopes greater than one (Figures 1, S7–S10).

The INLA models developed from the simulated samples may have had a more consistent predictive behavior with respect to increasing the size of the overall data set (i.e., have improved prediction when either the number of fish per sample event, or number of lakes increased) than REML models. This pattern was particularly apparent in the Walleye [Hg] data set, where increases in numbers of fish did not result in increased linear associations or correlations of predicted and measured values for models with 2–75 lakes (Figures S10 and S16), while INLA models generally had stronger linear associations and increased correlation with increases in number of fish for all sizes of lakes (Figures S10 and S16). This characteristic of INLA models means any addition of data to the training data set will benefit the overall INLA model, whereas REML models are only guaranteed to have increased performance with an increasing number of lakes and typically had good performance in models with 10–20 fish.

Each modeling approach had different benefits and drawbacks in terms of implementation. Based on the user experience during this study, the relative difficulty in application of each approach was REML < SER < MCMC < INLA. The REML approach was simple enough for a beginner R user to implement without requiring advanced programming knowledge. The SER approach required more programming knowledge to implement efficiently, and a significant amount of prescreening of data to ensure that a large enough span of fish sizes were included when developing a curve. Our approach was less time intensive, but less subjective than the involved model fitting of individual samples that are employed by some researchers performing SER. Our approach may have penalized the accuracy results of SER in our study, but would have also resulted in exclusion of more sample events from the SER analysis. There were more documentation and reading requirements to implement the MCMC and INLA models. Additionally, INLA, with the current INLA package implementation, poses a higher risk of incorrect implementation since the model formula input deviates from the typical R notation. There is thorough documentation of the model notation in the INLA package,^26^ but it is nonetheless a consideration in the implementation of this approach, as it added complexity when assessing the nested random effect of waterbody and sampling event.

Conceptually, the SER approach is the simplest to understand, given that it is essentially a group of independent linear models. However, using the SER approach to standardize the fish contaminant comparisons across lakes effectively removed some of the variation that is present in regional data sets, and thereby underestimates uncertainty. The REML, MCMC and INLA approaches account for this uncertainty in their model structure by allowing slopes and intercepts to vary by lake, and these mixed effect model (MEM) approaches quantify variation partitioning in the error term. MEMs are more conceptually challenging in terms of model specification, however, Bayesian models have more complexity compared than the standard REML approach. The complexity of the Bayesian (MCMC), and approximated Bayesian (INLA) approaches should be considered when model tuning is required, which is often required for smaller data sets (<300 fish). Relatively few studies^23,35,36^ have used Bayesian approaches to account for size effects, compared to more traditional linear modeling. This may be, in part, due to the lack of guidelines for best practices during implementation. For researchers pursuing predictive accuracy alone, the REML approach may be the best option due to its ease of implementation. Probability distributions from Bayesian models can inform sample size adjustments: variation around intercepts can provide information on sample size, and variation around slopes can indicate if the range of sites sampled is sufficient.

Mixed model approaches show promise for increasing the predictive power for estimating contaminant concentrations in fish by leveraging information from sampling events across time and space. We found that REML, MCMC, and INLA models of contaminant-size relationships were effective tools to predict contaminant concentrations of three fish species for two contaminants with different bioaccumulative potentials. Though the improvement on performance was more pronounced on smaller data sets, these techniques have more consistent performance across sample sizes than SER and are a good option when the bioaccumulative potential of a contaminant is unknown. REML was the most easily implemented approach but was outperformed by INLA for predicting samples with a small number of fish. REML also had a poorer model fit to the training data than INLA and MCMC, meaning it is less suitable for explaining the effects driving contaminant–size relationships. INLA generally was one of the less computationally intensive options and had less RMSE in the models, making it suitable for both explanatory and predictive applications. INLA can be performed quickly without high performance computing requirements. INLA also had comparable predictive accuracy at ∼12 or fewer samples to the full data set, which may allow for reductions in sampling effort for studies that intend to use a size-standardized contaminant concentration. Additionally, this approach allows for prediction in data sets that do not conform to regression assumptions (i.e., it allows for prediction for samples that do not have homogeneity of variance and lack balance in their sample sizes). In summary, we suggest that INLA is a suitable option for size-adjustment of contaminant concentrations in fish with the potential to increase overall sample sizes in lake-level studies. Use of these tools can enable greater power in investigations of important environmental drivers of contaminants in fish and increase the number of lakes with consumption guidelines in fish consumption advisory programs. A future avenue of research may be investigating the strengths of each modeling approach in different scenarios, to evaluate which models are most appropriate for prediction and which are better at analyzing trends at various effect sizes. We have provided example code with this paper that may assist in those efforts or help researchers interested in using INLA for size standardizing contaminant concentrations.

## Data Availability

The processed data, scripts used to create this manuscript, and the INLA example code are available for download at the public repository managed by the WETlab at the Great Lakes Forestry Centre: https://github.com/GLFC-WET/HGAS_master. The data used for this paper is also deposited at the zenodo repository located at 10.5281/zenodo.13835461.

## Supporting Information Available

Summary of test and training data sets, graphical summary of data distribution and conceptual handling of data, alternative graphical representation of results, additional RMSE results, and simulated sampling results (PDF)
