# Supplementary material for: Mixed Model Approaches Can Leverage Database Information to Improve the Estimation of Size-Adjusted Contaminant Concentrations in Fish Populations
Source: Environ Sci Technol. 2025 Mar 5;59(10):4797–806. doi: 10.1021/acs.est.4c10303 (PMC11924241; doi:10.1021/acs.est.4c10303)
Supplement: Supplementary file 1 — es4c10303_si_001.pdf [file es4c10303_si_001.pdf]

Supplemental Information

Supplemental tables and figures for the manuscript "Mixed model approaches can leverage database information to improve the estimation of size-adjusted contaminant concentrations in fish populations"

* Emily Smenderovac (corresponding author), Great Lakes Forestry Centre, Natural Resources Canada, P6A 2E5,, *emily.smenderovac@nrcan-rncan.gc.ca*
Brian W. Kielstra, Ecometrix, Guelph, Ontario, Great Lakes Forestry Centre, Natural Resources Canada, P6A 2E5, *bkielstra@ecometrix.ca*
Calvin Kluke, Vale Living with Lakes Centre, Laurentian University, P3E 2C6, *calvin.kluke1@gmail.com*
Thomas A. Johnston, Vale Living with Lakes Centre, Ontario Ministry of Natural Resources, P3E 2C6, *tom.johnston@ontario.ca*
Satyendra P. Bhavsar, Ontario Ministry of the Environment, Conservation and Parks, Ontario M9P 3V6, *satyendra.bhavsar@ontario.ca*
Robert Mackereth, Centre for Northern Forest Ecosystem Research, Ontario Ministry of Natural Resources and Forestry, P7B 5E1, *rob.mackereth@ontario.ca*
Stephanie Melles, Department of Chemistry and Biology, Urban Water Research Centre, Toronto Metropolitan University, M5B 2K3, *stephanie.melles@torontomu.ca*
Gretchen L. Lescord, Forests Fisheries and Geomatic Sciences, University of Florida, 32611, *lescord.g@ufl.edu*
Erik J.S. Emilson, Great Lakes Forestry Centre, Natural Resources Canada, P6A 2E5, *erik.emilson@nrcan-rncan.gc.ca*

Summary:

17 pages

1 table

16 Figures

Supplemental Tables

Table S1: Summary of number fish, and number of sample events for each contaminant and species in the dataset used in this analysis. Values are listed as 'train fish (train sample events) / test fish (test sample events)' for each dataset.

CONTAMINANT	SPECIES_NAME	SER	REML	boot_REML	MCMC	INLA
As	Lake Trout	65(10)/10(7)	120(27)/10(7)	120(27)/10(7)	120(27)/10(7)	120(27)/10(7)
As	Northern Pike	156(19)/47(33)	465(104)/50(35)	465(104)/50(35)	465(104)/50(35)	465(104)/50(35)
As	Walleye	152(21)/40(29)	416(98)/43(30)	416(98)/43(30)	416(98)/43(30)	416(98)/43(30)
Hg	Lake Trout	4234(396)/558(312)	6122(638)/559(313)	6122(638)/559(313)	6122(638)/559(313)	6122(638)/559(313)
Hg	Northern Pike	10095(1017)/1620(854)	13870(1474)/1625(859)	13870(1474)/1625(859)	13870(1474)/1625(859)	13870(1474)/1625(859)
Hg	Walleye	14246(1172)/2551(1043)	17931(1445)/2555(1047)	17931(1445)/2555(1047)	17931(1445)/2555(1047)	17931(1445)/2555(1047)

Supplemental Figures

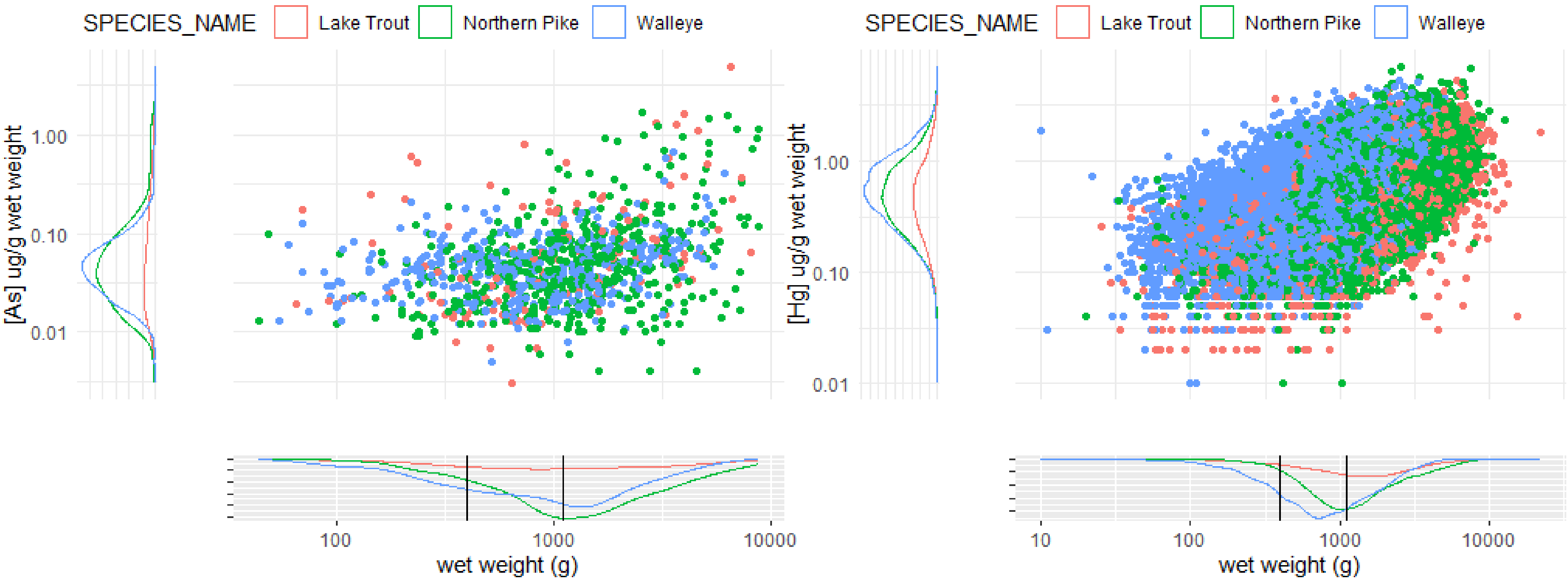

Figure S1: Number of fish at different body weights and contaminant concentrations for [As] and [Hg]. Vertical black lines show the weights that are commonly used for standardization.

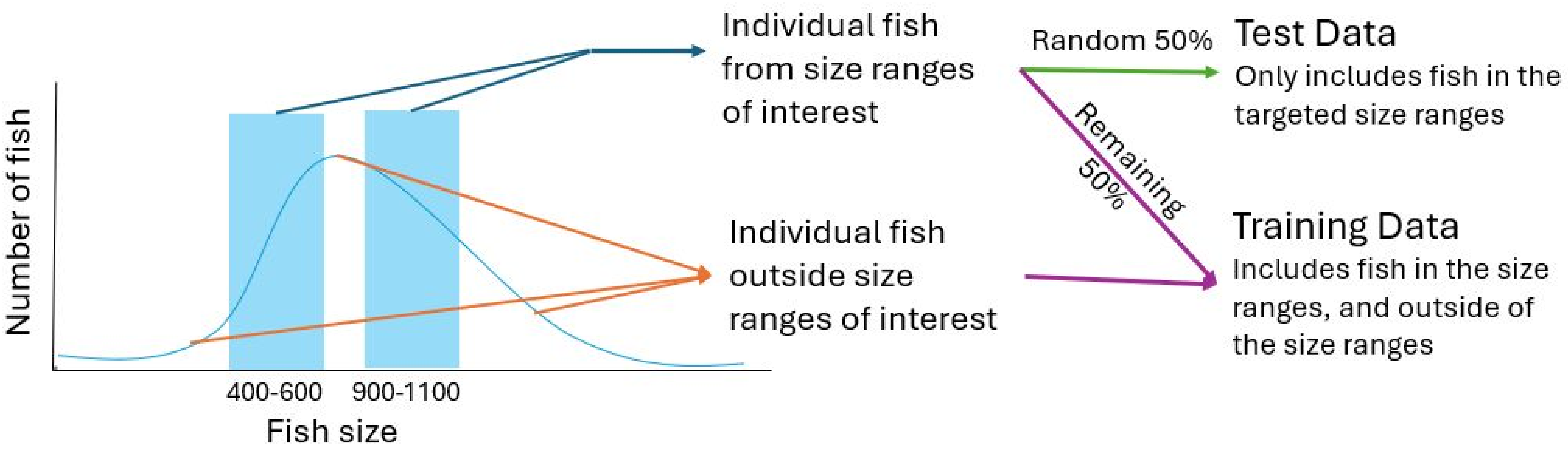

Figure S2: Conceptual disgram of testing and training dataset construction for each fish species. The figure shows the theoretical distribution of fish sizes as a blue line representing a density distribution, the selection of targeted sizes as blue rectangles and then a flow diagram showing the different data splits.

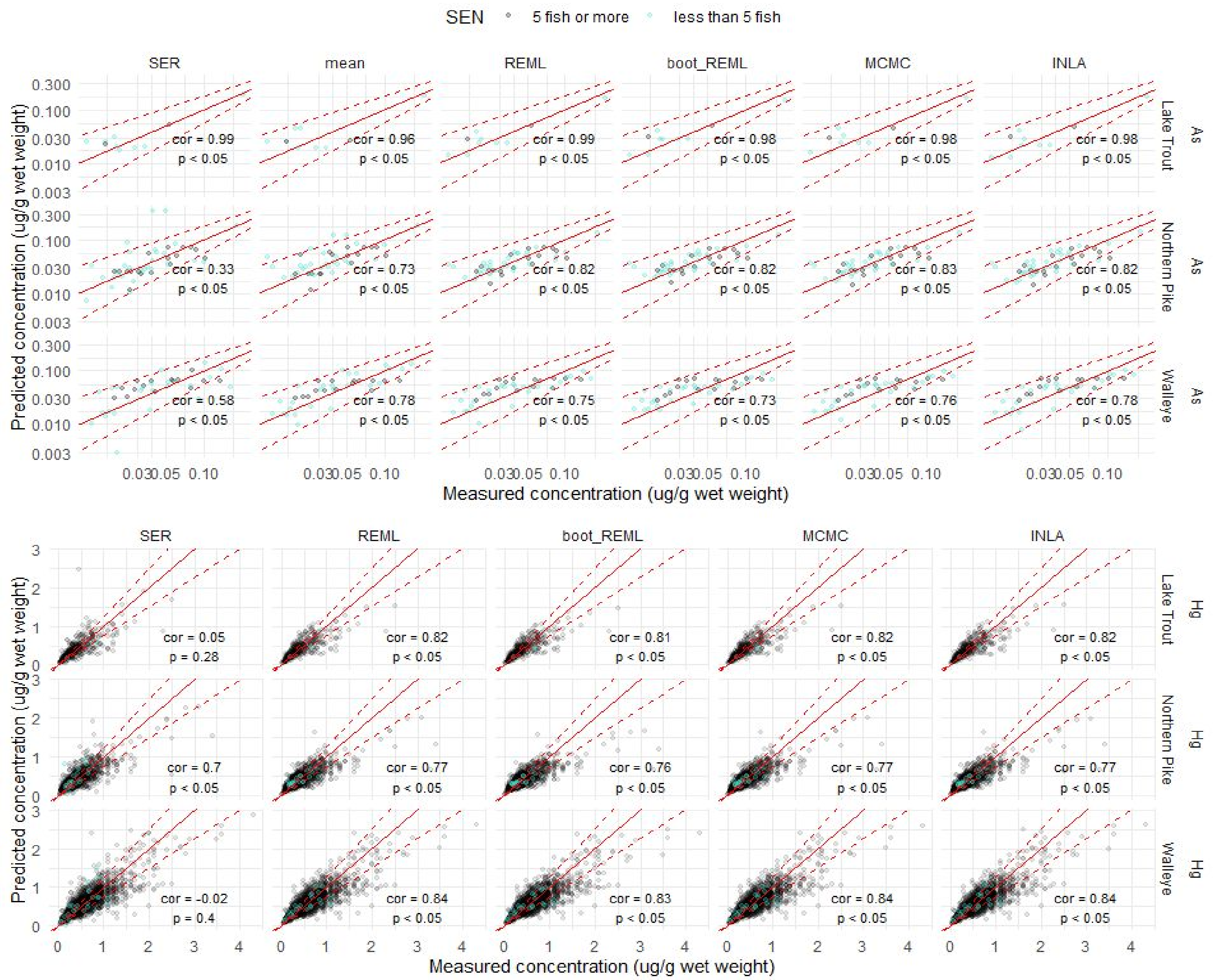

Figure S3: Predicted concentration of [As] and [Hg] in tissue of 400-600g and 900-1100g fish compared to measured values. Points display each individual fish prediction (with blue points representing predictions at sampling events with less than 5 fish, and black points representing predictions from sampling events with at least 5 fish) and a solid red line shows a 1-1 relationship. The dashed red lines provide an interval for predicted values that are within 25% of the measured value. Correlation statistics are included where there were enough values to compute them. Axes are shown on a log scale, as values were modelled from log-transformed values.

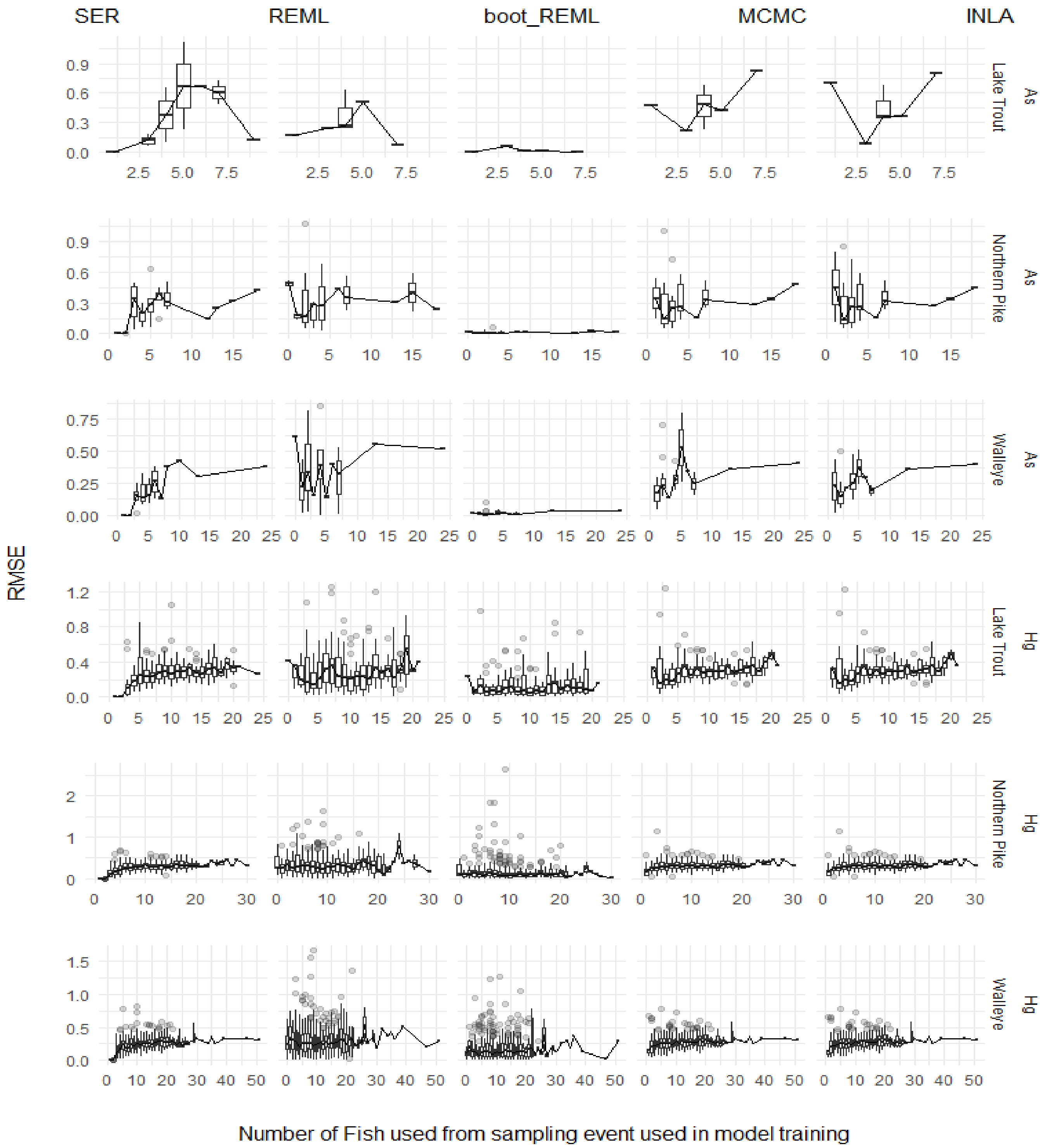

Figure S4: Root mean squared error (RMSE) of models from sampling events trained with different numbers of fish. RMSE of training set sampling events are compared to the number of fish used from the sampling event that were included in the training dataset. For each N, a boxplot displays the 25th and 75th percentiles and the median RMSE, with the whiskers extending out to 1.5 x the interquartile range. A line is plotted across the median values to show the overall trend in the values.

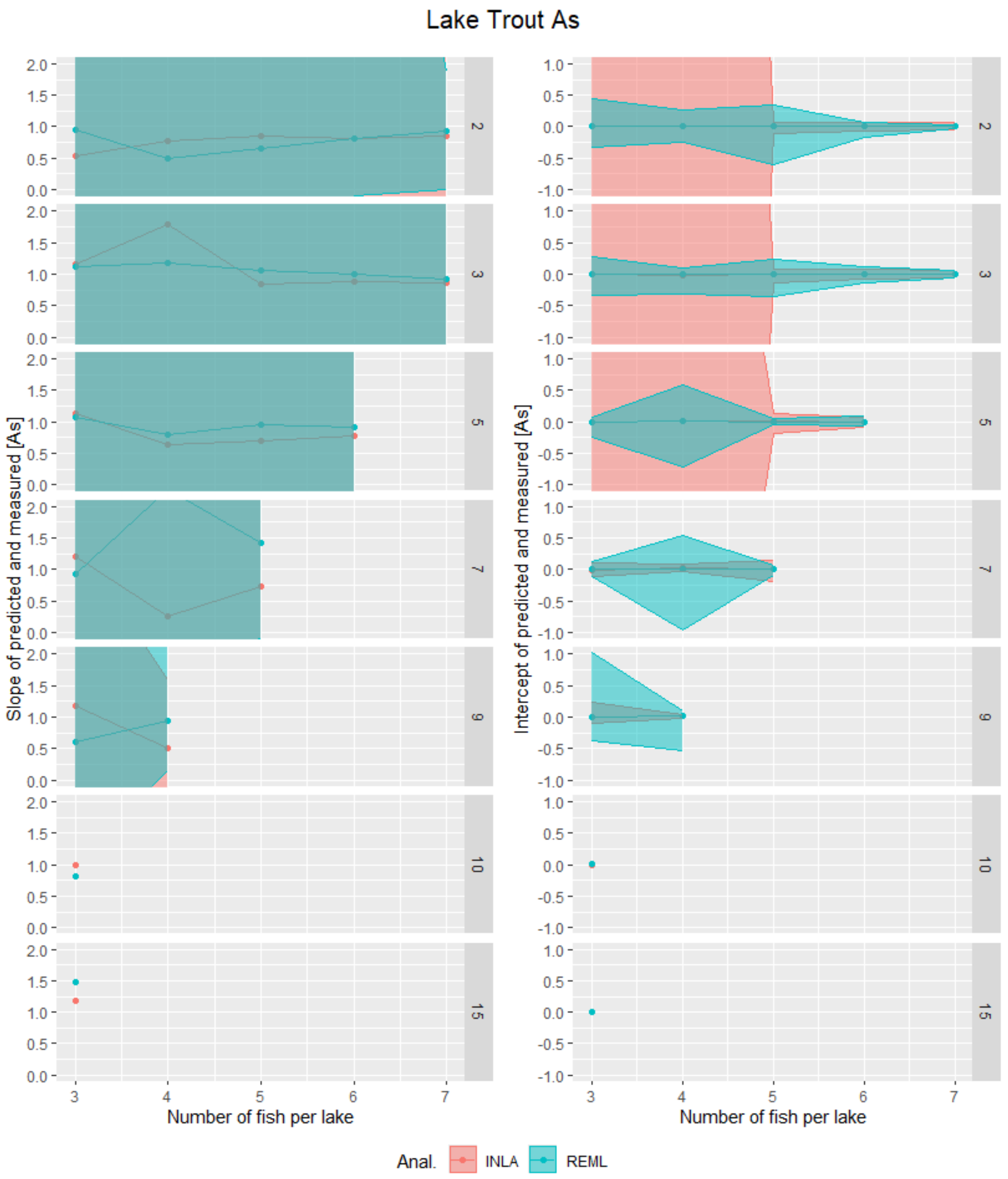

Figure S5: Range of slopes and intercepts for simulated samplings of Lake Trout [As] INLA and REML predictions from different lake and fish number combinations. Maximum and minimum slopes and intercepts from the 95% confidence intervals for each fish number (x-axis) and lake number (vertical panels) combination are represented by a shaded area and the median slope or intercept for each simulations are represented as a central line with points. INLA and REML results are distinguished by colour.

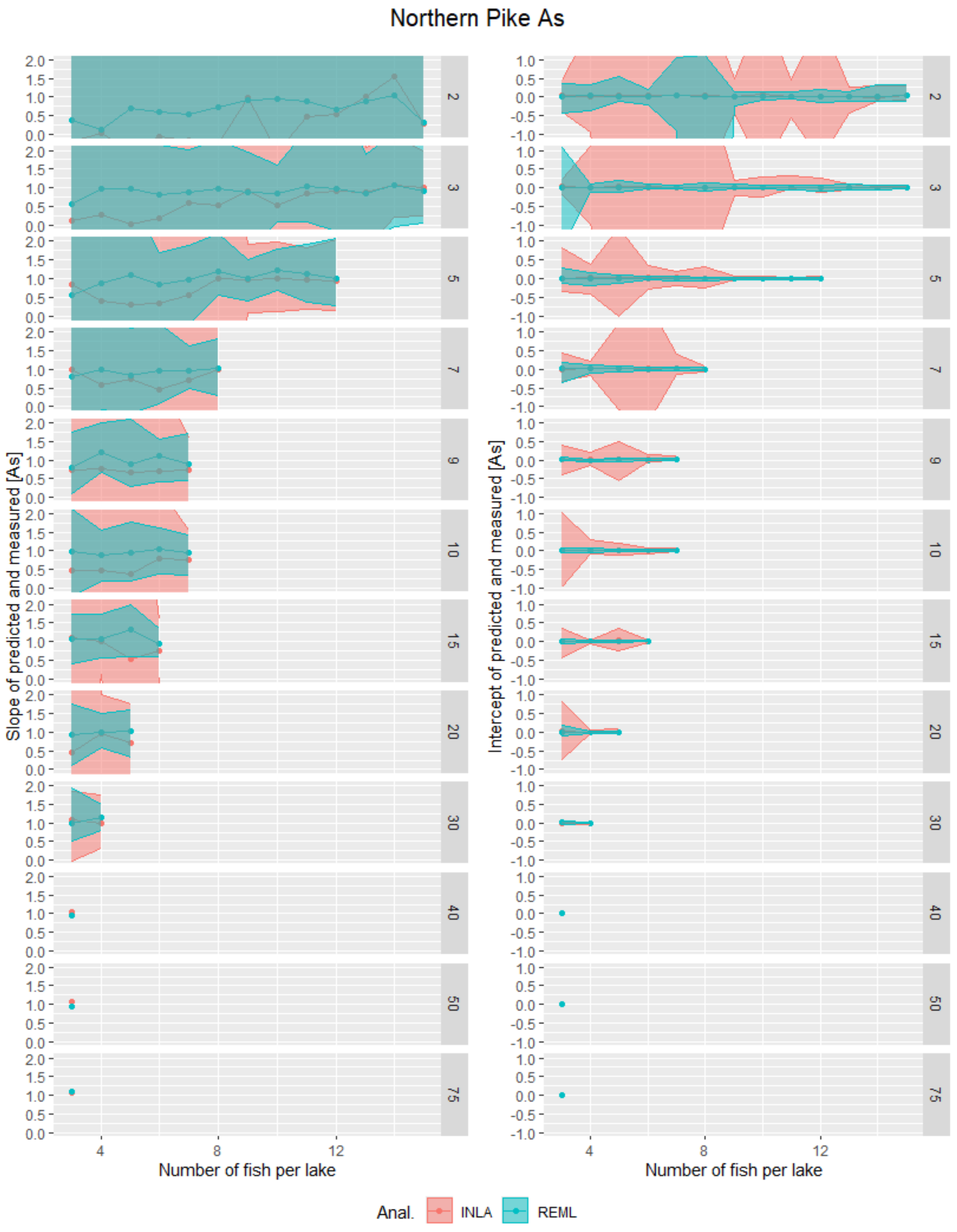

Figure S6: Range of slopes for simulated samplings of Northern Pike [As] INLA and REML predictions from different lake and fish number combinations. Maximum and minimum slopes and intercepts from the 95% confidence intervals for each fish number (x-axis) and lake number (vertical panels) combination are represented by a shaded area and the median slope or intercept for each simulations are represented as a central line with points. INLA and REML results are distinguished by colour.

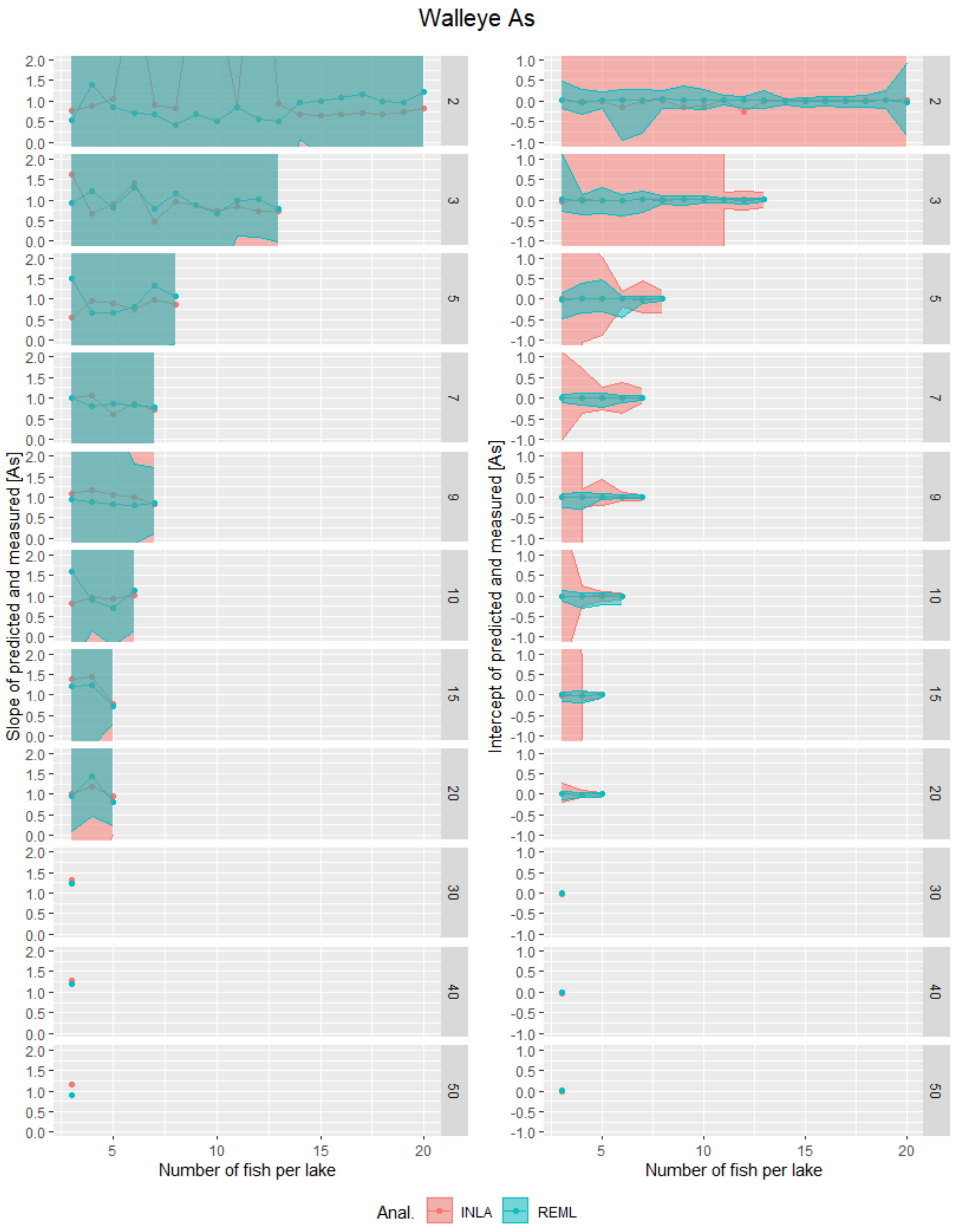

Figure S7: Range of slopes for simulated samplings of Walleye [As] INLA and REML predictions from different lake and fish number combinations. Maximum and minimum slopes and intercepts from the 95% confidence intervals for each fish number (x-axis) and lake number (vertical panels) combination are represented by a shaded area and the median slope or intercept for each simulations are represented as a central line with points. INLA and REML results are distinguished by colour.

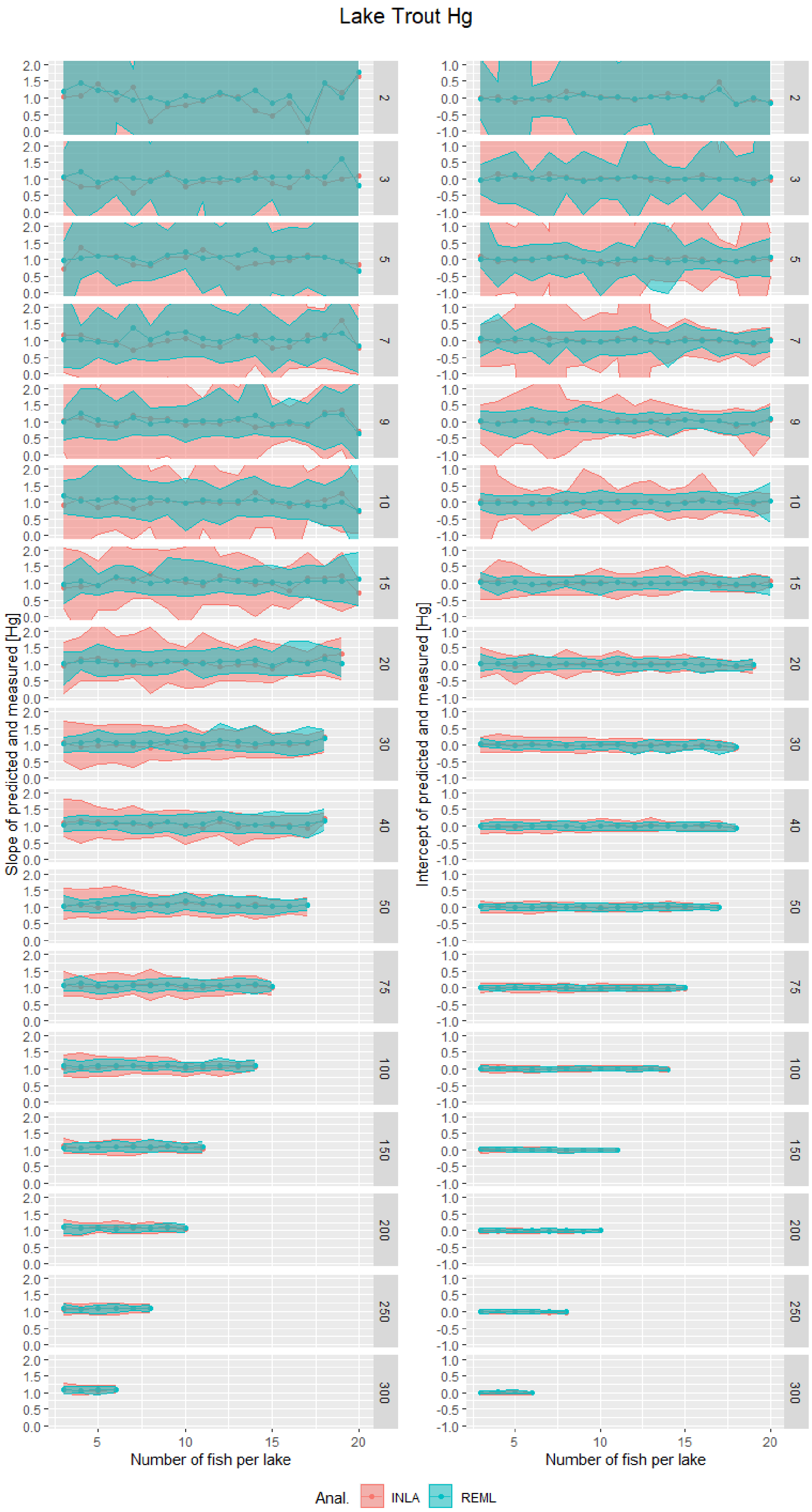

Figure S8: Range of slopes for simulated samplings of Lake Trout [Hg] INLA and REML predictions from different lake and fish number combinations. Maximum and minimum slopes and intercepts from the 95% confidence intervals for each fish number (x-axis) and lake number (vertical panels) combination are represented by a shaded area and the median slope or intercept for each simulations are represented as a central line with points. INLA and REML results are distinguished by colour.

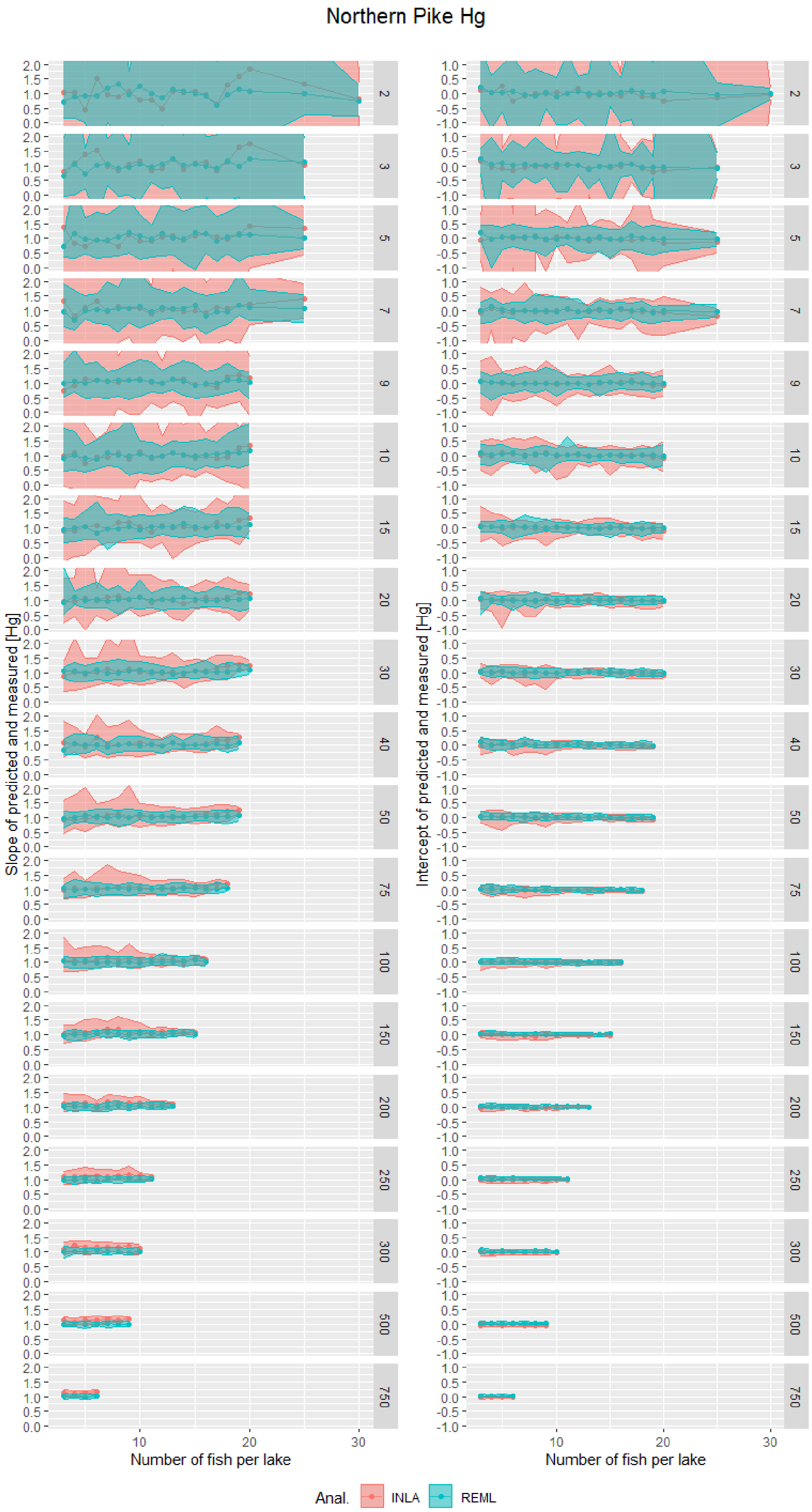

Figure S9: Range of slopes for simulated samplings of Northern Pike [Hg] INLA and REML predictions from different lake and fish number combinations. Maximum and minimum slopes and intercepts from the 95% confidence intervals for each fish number (x-axis) and lake number (vertical panels) combination are represented by a shaded area and the median slope or intercept for each simulations are represented as a central line with points. INLA and REML results are distinguished by colour.

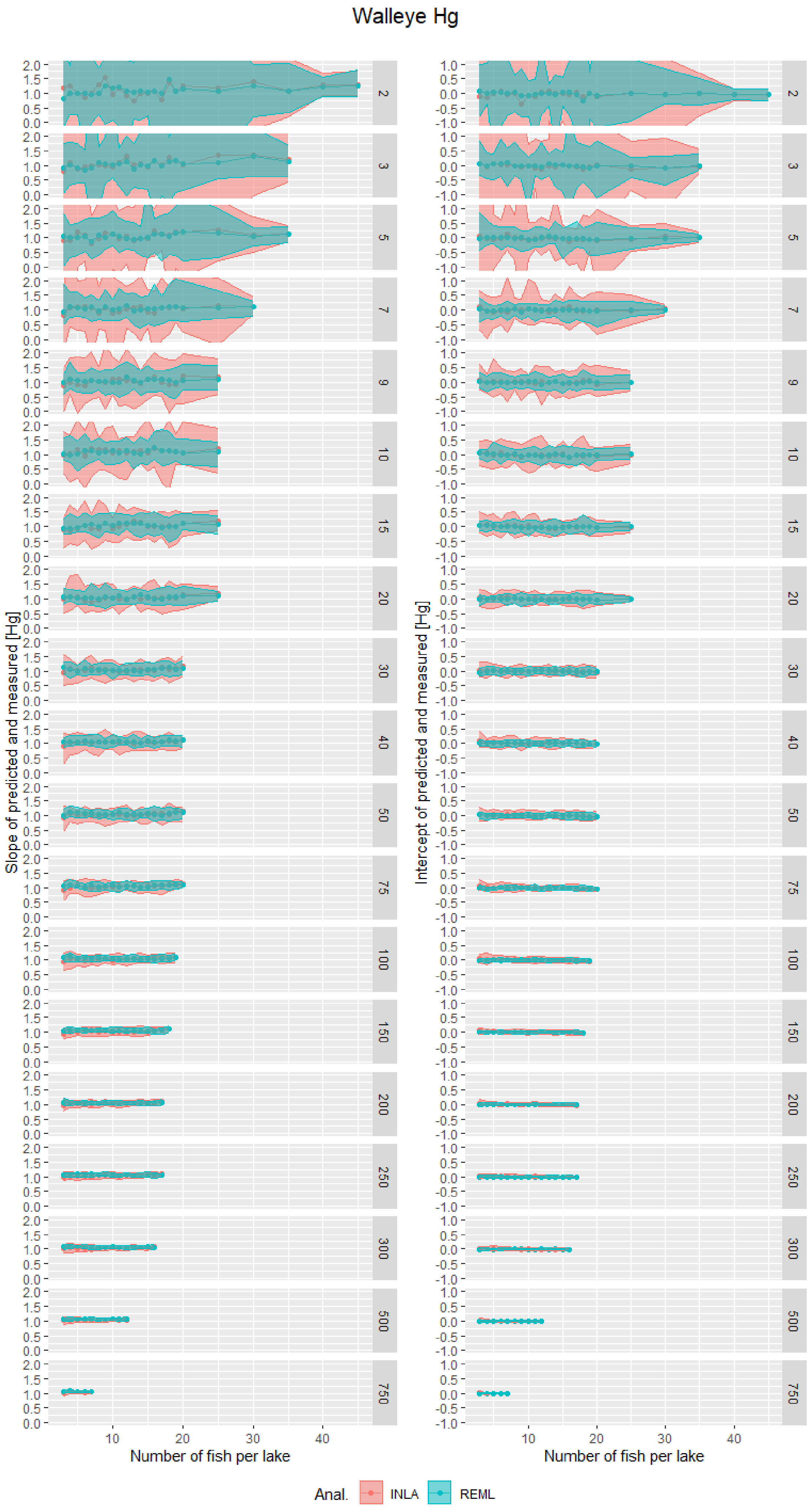

Figure S10: Range of slopes for simulated samplings of Walleye [Hg] INLA and REML predictions from different lake and fish number combinations. Maximum and minimum slopes and intercepts from the 95% confidence intervals for each fish number (x-axis) and lake number (vertical panels) combination are represented by a shaded area and the median slope or intercept for each simulations are represented as a central line with points. INLA and REML results are distinguished by colour.

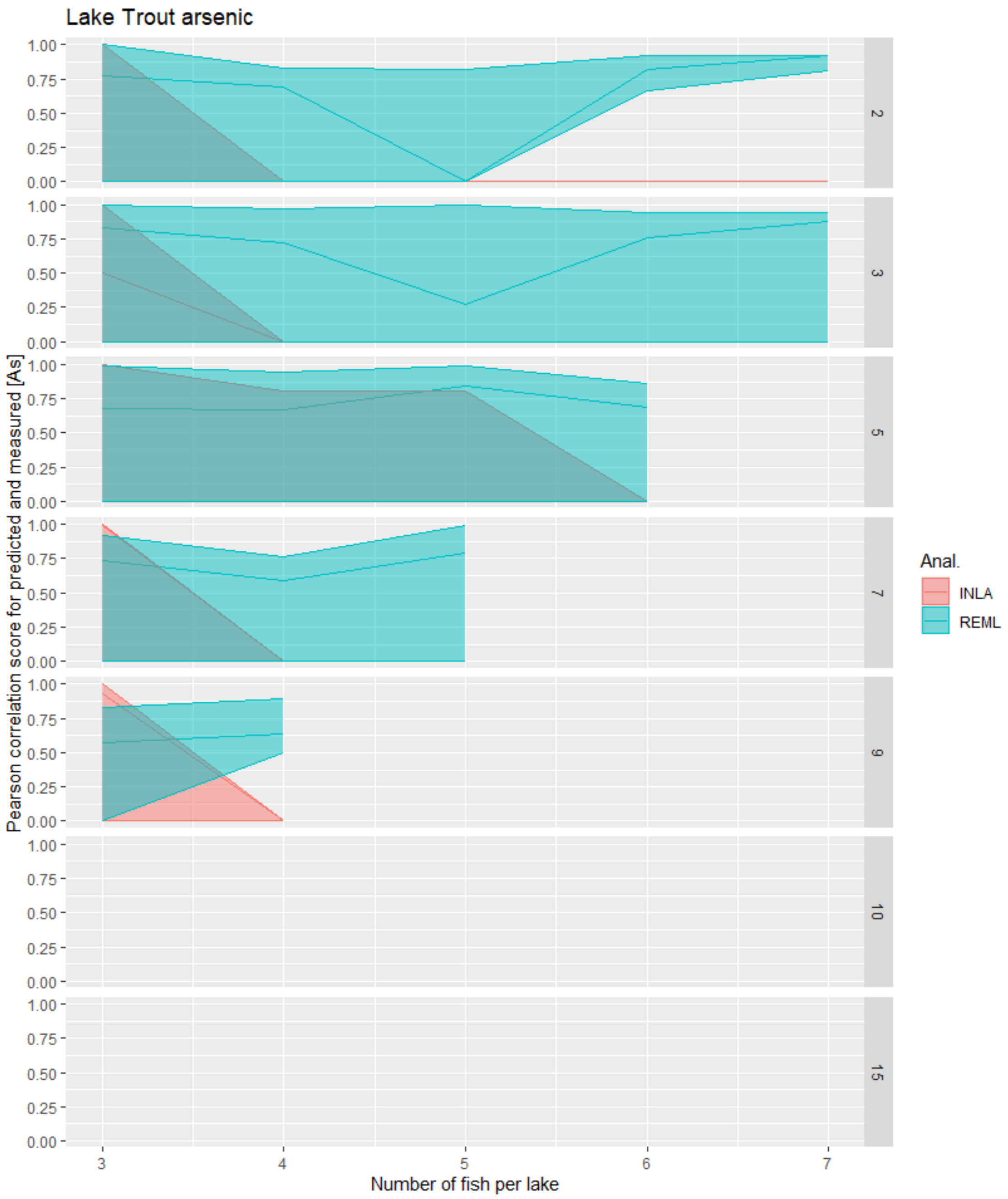

Figure S11: Range of Pearson correlation scores for simulated samplings of Lake Trout [As] INLA and REML predictions from different lake and fish number combinations. Maximum and minimum correlations for each fish number (x-axis) and lake number (vertical panels) combination are represented by a shaded area and the median correlation for the simulations are represented as a central line. INLA and REML results are distinguished by colour.

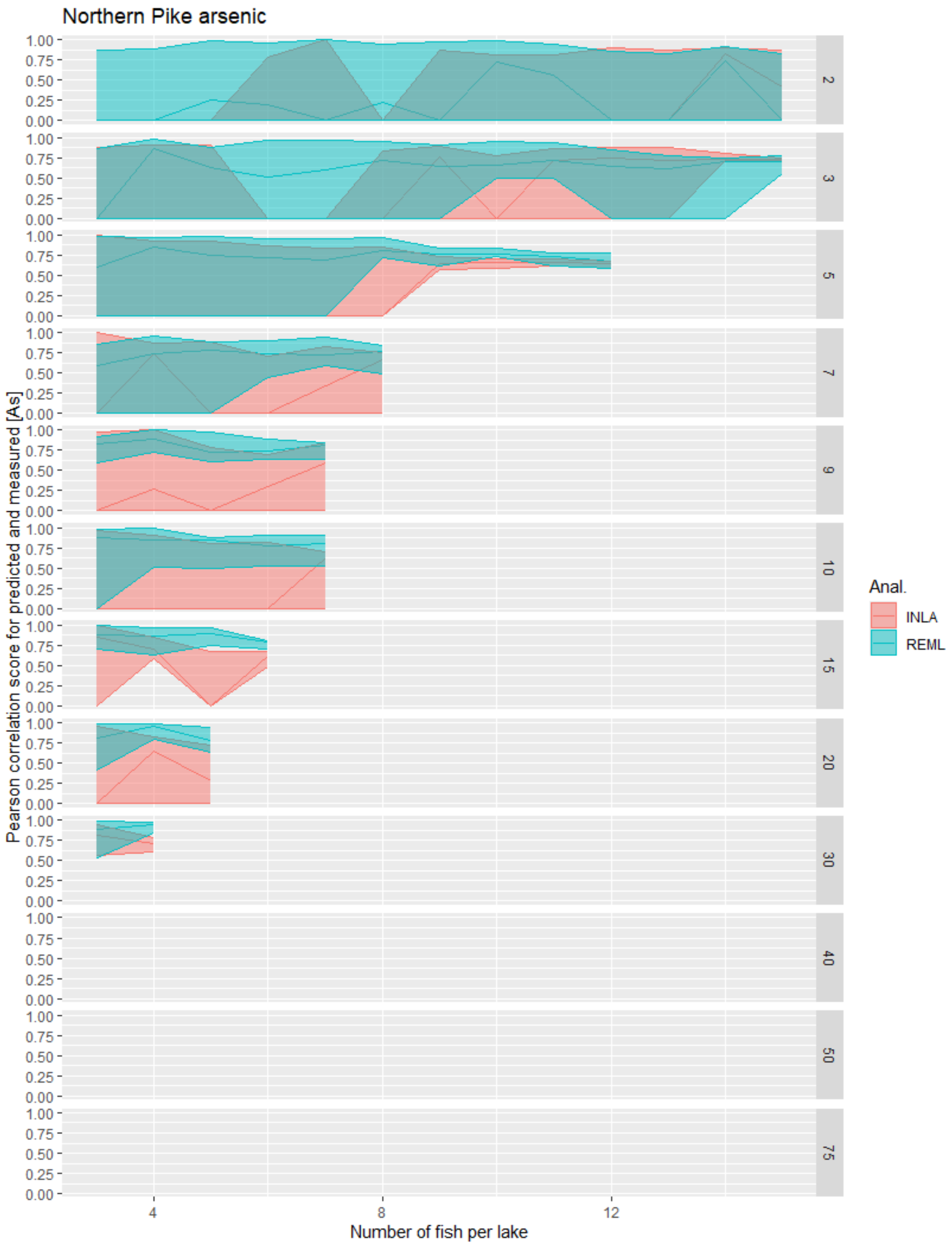

Figure S12: Range of Pearson correlation scores for simulated samplings of Northern Pike [As] INLA and REML predictions from different lake and fish number combinations. Maximum and minimum correlations for each fish number (x-axis) and lake number (vertical panels) combination are represented by a shaded area and the median correlation for the simulations are represented as a central line. INLA and REML results are distinguished by colour.

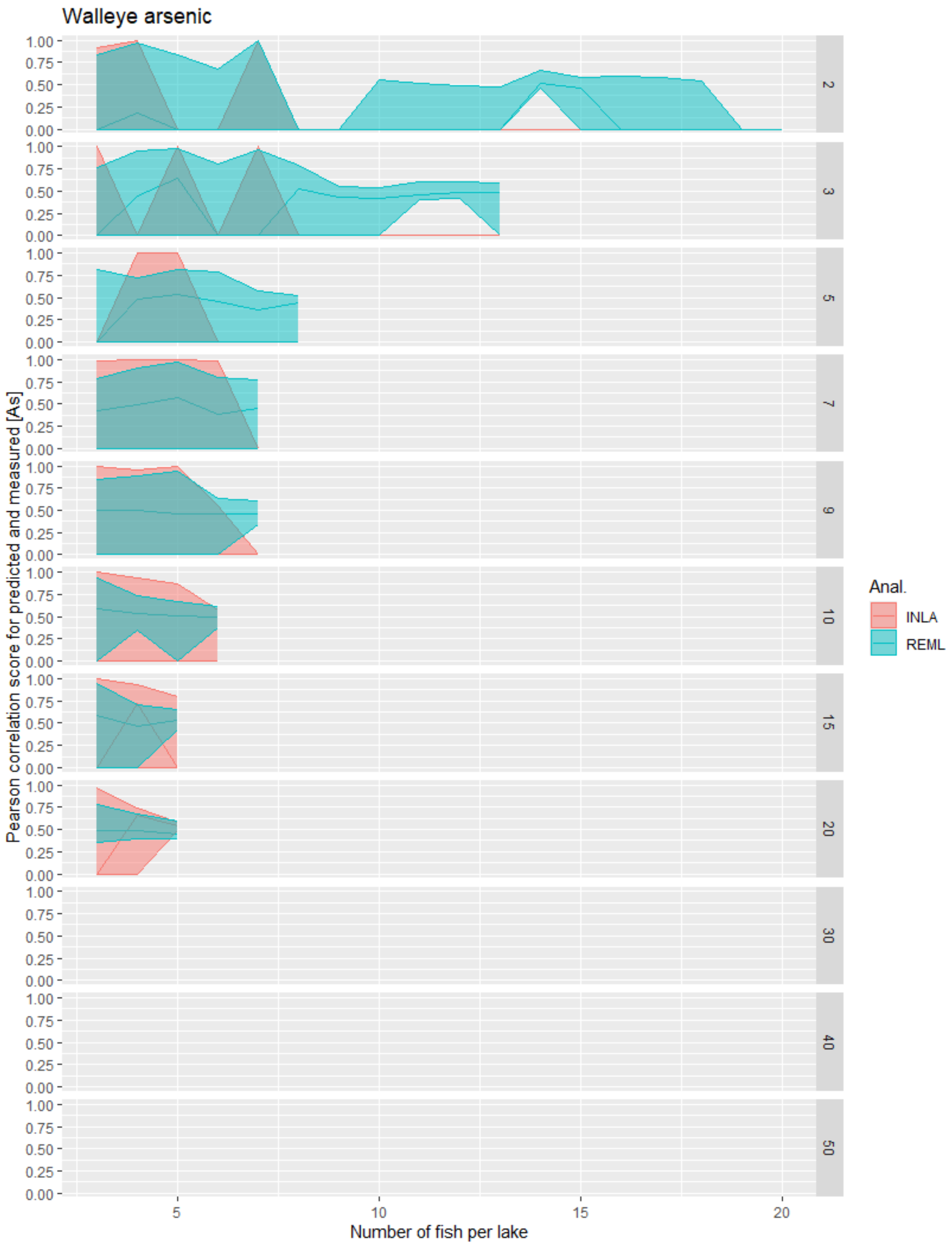

Figure S13: Range of Pearson correlation scores for simulated samplings of Walleye [As] INLA and REML predictions from different lake and fish number combinations. Maximum and minimum correlations for each fish number (x-axis) and lake number (vertical panels) combination are represented by a shaded area and the median correlation for the simulations are represented as a central line. INLA and REML results are distinguished by colour.

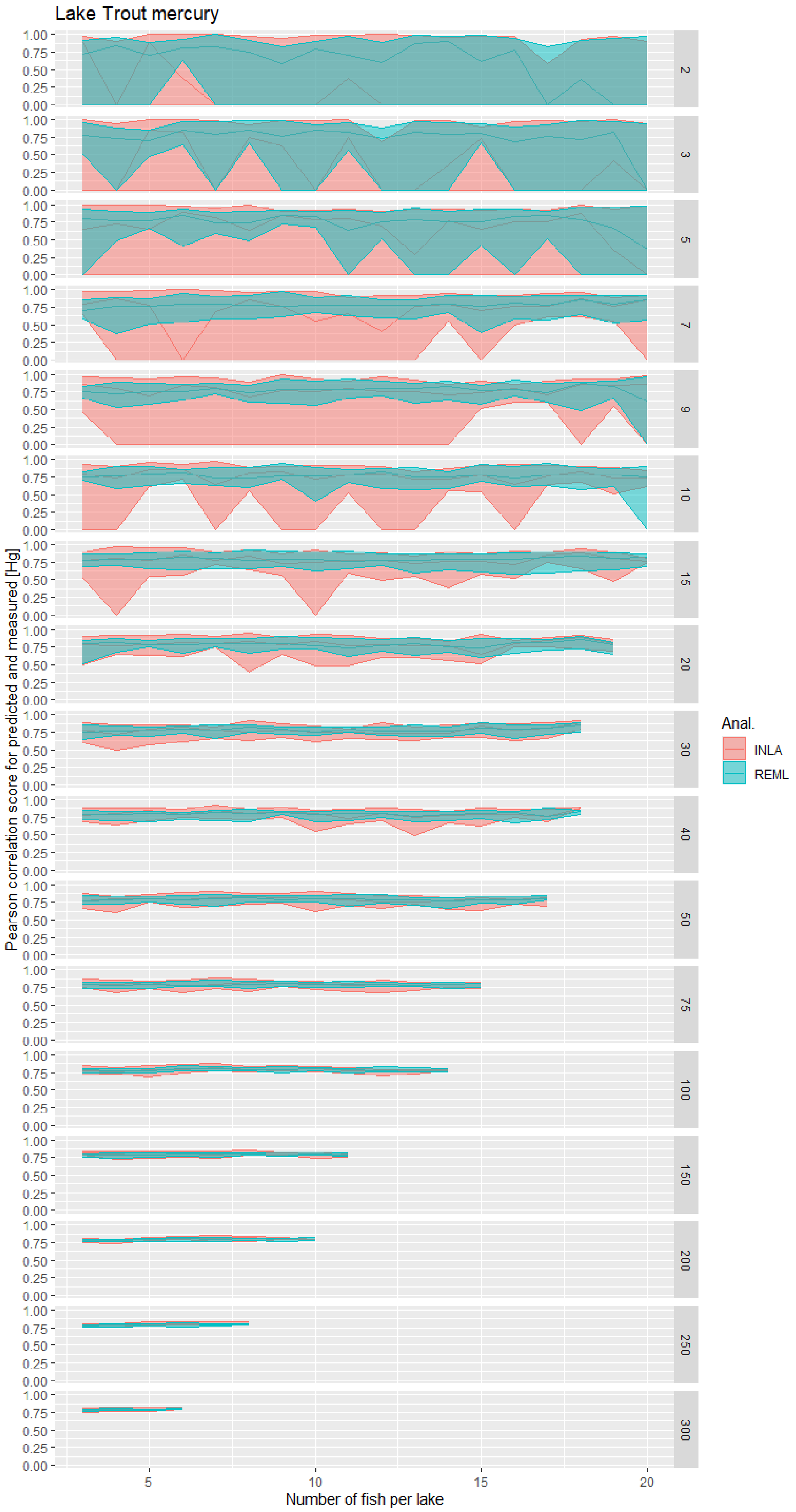

Figure S14: Range of Pearson correlation scores for simulated samplings of Lake Trout [Hg] INLA and REML predictions from different lake and fish number combinations. Maximum and minimum correlations for each fish number (x-axis) and lake number (vertical panels) combination are represented by a shaded area and the median correlation for the simulations are represented as a central line. INLA and REML results are distinguished by colour.

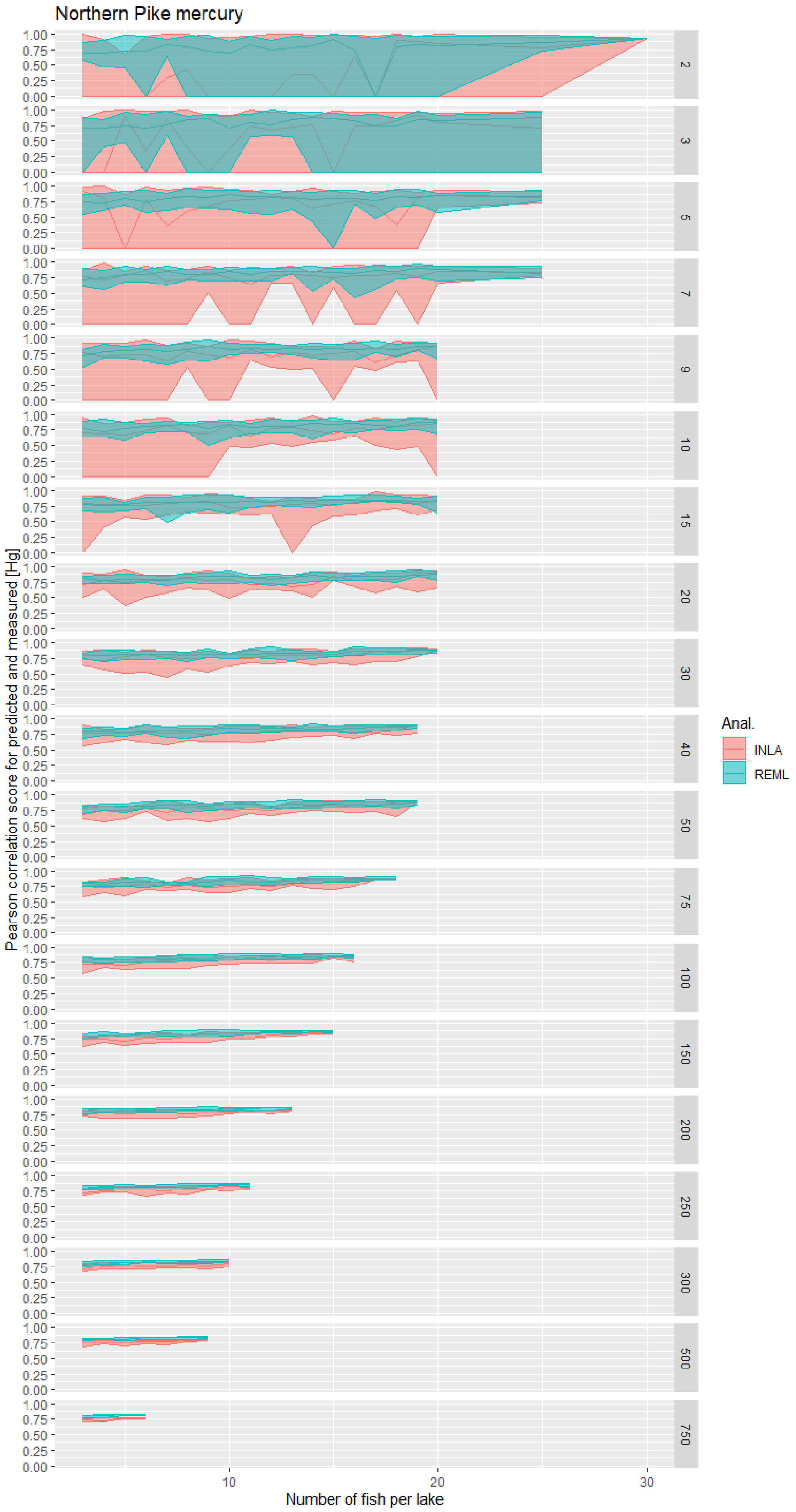

Figure S15: Range of Pearson correlation scores for simulated samplings of Northern Pike [Hg] INLA and REML predictions from different lake and fish number combinations. Maximum and minimum correlations for each fish number (x-axis) and lake number (vertical panels) combination are represented by a shaded area and the median correlation for the simulations are represented as a central line. INLA and REML results are distinguished by colour.

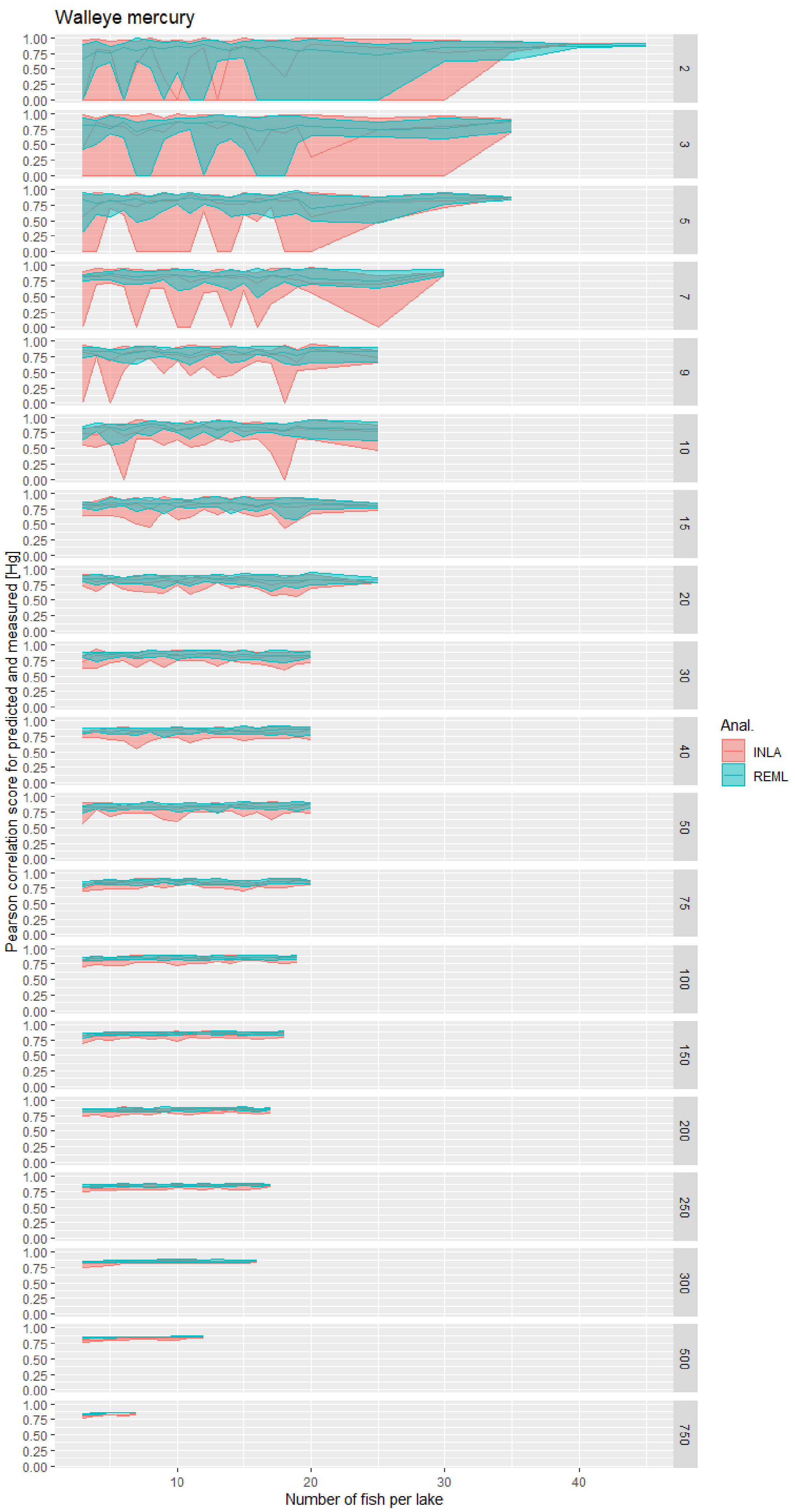

Figure S16: Range of Pearson correlation scores for simulated samplings of Walleye [Hg] INLA and REML predictions from different lake and fish number combinations. Maximum and minimum correlations for each fish number (x-axis) and lake number (vertical panels) combination are represented by a shaded area and the median correlation for the simulations are represented as a central line. INLA and REML results are distinguished by colour.
